# Diagonal Method to Measure Synergy Among Any Number of Drugs

**DOI:** 10.3791/57713

**Published:** 2018-06-21

**Authors:** Melike Cokol-Cakmak, Feray Bakan, Selim Cetiner, Murat Cokol

**Affiliations:** ^1^Faculty of Engineering and Natural Sciences, Sabanci University; ^2^Nanotechnology Research and Application Center, Sabanci University; ^3^Laboratory of Systems Pharmacology, Harvard Medical School

**Keywords:** Biology, Issue 136, Drug interactions, checkerboard assay, drug synergy, drug combinations, Loewe additivity model, high-order drug interactions

## Abstract

A synergistic drug combination has a higher efficacy compared to the effects of individual drugs. Checkerboard assays, where drugs are combined in many doses, allow sensitive measurement of drug interactions. However, these assays are costly and do not scale well for measuring interaction among many drugs. Several recent studies have reported drug interaction measurements using a diagonal sampling of the traditional checkerboard assay. This alternative methodology greatly decreases the cost of drug interaction experiments and allows interaction measurement for combinations with many drugs. Here, we describe a protocol to measure the three pairwise interactions and one three-way interaction among three antibiotics in duplicate, in five days, using only three 96-well microplates and standard laboratory equipment. We present representative results showing that the three-antibiotic combination of Levofloxacin + Nalidixic Acid + Penicillin G is synergistic. Our protocol scales up to measure interactions among many drugs and in other biological contexts, allowing for efficient screens for multi-drug synergies against pathogens and tumors.

**Figure:**
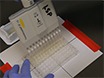


## Introduction

Drug combinations may exhibit surprisingly high or low effect on a phenotype given the effects of constituent drugs, corresponding to synergistic or antagonistic drug interactions, respectively^1^,^2^,^3^. The use of synergistic combinations may allow dose-escalation for efficacy increase and dose-reduction for side-effect relief. Combination treatments may also apply multiple setbacks to cellular machinery, thereby blocking potential evolutionary escape mechanisms to resistance^4^. Therefore, combinations of three or more drugs are routinely used in pathogen or cancer treatment^5^.

Synergy and antagonism are defined by a comparison between the observed effect of a combination versus an expected effect given individual drug effects. Among the models for drug interactions, Loewe additivity is the most stringent and has a well-defined null model **(**Figure 1**)**^6^, and the inferred synergy/antagonism interaction is independent of the drug concentration used^6^,^7^. However, the Loewe model is experimentally costly even for a pairwise interaction test. Drug interaction assays traditionally comprise of a 2D matrix of drug concentration combinations (a checkerboard assay) **(**Figure 2**)**. If 5 doses are used for each drug, then 25 combinations are required, corresponding to one half of a microplate if experiments are conducted in replicate. The cost of this approach prohibits synergy measurement by the Loewe additivity model for multi-drug combinations **(**Figure 3**)**. For example, to test a 10-way interaction, traditional methods would require more than 100 thousand microplates, barring experimental measurement of high-order synergies by the stringent, well-theorized and concentration-independent Loewe additivity model^8^.

Current clinical treatments utilize only a fraction of possible drug combinations. For example, the standard treatment of active tuberculosis is a combination of three antibiotics. There are approximately 20 antibiotics used in *Mycobacterium tuberculosis *(*Mtb*) treatment. There are 1140 possible 3-way combinations among 20 drugs, each with the potential to have a strong synergy against *Mtb*. As there has been no cost-effective method to measure drug interactions among many drugs, potentially life-saving synergistic combinations remain untested.

Here, we describe a simple protocol to measure pairwise and three-way drug interactions by sampling only the diagonal of a checkerboard assay **(**Figure 4 and****Figure 5**)**. The underlying concept of sampling the diagonal of a checkerboard experiment was theorized by Berenbaum in his seminal work in 1978^9^. Yet, this approach has only recently been applied to drug synergy screens^10^,^11^,^12^. We present our protocol with *Escherichia coli (E. coli*) and the growth phenotype. However, we note that the protocol is independent of the biological species and phenotype of interest, and hence may be applied to the measurement of high-order drug synergy in other biological contexts.

## Protocol

NOTE: Any small molecule that inhibits *E. coli* bacteria growth can be used for the diagonal method. In this protocol, levofloxacin (LEV), nalidixic acid (NAL), and Penicillin G (PNG) will be used as an example, since these drugs show a salient three-way synergy. The workflow of this protocol is shown as Figure 6. Carry out all steps at room temperature. Use fresh aliquots of bacteria and drugs each day. Perform the experiment under biosafety levels appropriate for *E. coli*.

### 1. Preparation Steps

1. Prepare Luria-Bertani (LB) media by adding 25 g of LB broth to 1 L of distilled water and mix. Autoclave at 121 °C for 15 min and store the autoclaved media at room temperature.
2. Prepare *E. coli *glycerol stocks by mixing equal volumes of sterile 50% glycerol and bacterial cells diluted to OD_600_ = 1 in LB broth and freeze 150 µL aliquots in 1.5 mL microcentrifuge tubes at -80 °C.
3. Dissolve 20 mg of antibiotics, LEV, NAL and PNG in 1 mL of dimethyl sulfoxide (DMSO) each. Dilute LEV solution 100x to 0.2 µg/mL by mixing 10 µL of LEV solution with 990 µL of DMSO. Use 0.2 mg/mL LEV, and 20 mg/mL NAL and PNG in the proceeding steps.
4. Aliquot 50 µL of each antibiotic to 1.5 mL microcentrifuge tubes and freeze at -20 °C.
5. Take one 150 µL aliquot of *E. coli* from -80 °C. Thaw.
6. Add 100 µL of *E. coli* glycerol stock in 5 mL of LB media in a 14 mL culture tube.
7. Shake the tubes in a 37 °C incubator overnight at 200 rpm.

### 2. Serial Dilution Dose-Response Experiment

1. Take one aliquot of drugs LEV, NAL and PNG from -20 °C, leave them at room temperature for 10 min to thaw and prepare for serial dilutions of these drugs.
2. Prepare 1100 µL of LB-10%sol by mixing 990 µL of LB media and 110 µL of solvent (DMSO).
3. Prepare 500 µL of LB-10%LEV by mixing 450 µL of LB media and 50 µL of LEV.
4. Vortex LB-10%sol for 5 s at the highest setting. Add 20 µL of LB-10%sol to the top four rows of wells in a 96-well microplate.
5. Vortex LB-10%LEV for 5 s at the highest setting. Add 20 µL of LB-10%LEV to the first well in row A.
6. Prepare two-fold serial dilution for LB-10%LEV by taking 20 µL from the first well, adding to the second well, pipetting up and down five times. Repeat this for all wells sequentially until the 11th well, which ends up with 40 µL (Figure 7A).
7. Remove and discard 20 µL of the content from the 11th well, using a micropipette.
8. Repeat steps 2.3-2.7 for the drugs NAL and PNG using the second and third rows of the microplate, respectively.
9. Repeat steps 2.3-2.7 for the drug LEV again on fourth row as an internal positive control.
10. Using a spectrophotometer, measure the OD_600_ of a 1:10 dilution of the culture (steps 1.5-1.7).
11. Dilute the cells in 5 mL of LB media to an OD_600_ of 0.01. Pour into a reservoir.
12. Using a multichannel micropipette, add 80 µL of the diluted cells to the drug serial dilutions prepared in step 2.4-2.9. The final drug concentrations in each well is shown in Figure 7A. Seal the plate to prevent evaporation.
13. Incubate the plate for 16 h at 37 °C.
14. Start a new bacterial culture to use in step 3 (repeat steps 1.5-1.7).

### 3. Linear Dilution Dose-Response Experiment

1. Measure the OD_600_ absorbance for serial dilution dose-response plate from step 2 using a plate reader (Figure 7A** right**) and interpret the results based on the following steps.
2. Normalize the growth by dividing the growth in each well with the growth in the no drug control for each row and compute percent growth by normalizing OD_600_to no drug condition.
3. For each drug, locate the wells that have ~50% growth inhibition (IC50), shown in orange in Figure 7A** right**. Assign the concentration in these wells as "serial IC50" for each drug.
4. Thaw fresh drug aliquots, prepare 1 mL of LB-10%sol by mixing LB media and solvent (DMSO) in a 9:1 ratio and LB-10%drug by mixing LB media and drug in a 9:1 ratio, where the drug's concentration is 100x of each drug's serial IC50 before adding the LB media, as chosen at step 3.3.
5. Prepare linearly increasing doses of drugs LEV, NAL and PNG in 11 concentrations, by mixing LB-10%drug and LB-10%sol in volumes shown in Figure 7B.
6. Prepare linearly increasing doses of LEV on fourth row as an internal positive control.
7. Measure the OD_600_ of the 1:10 dilution of the culture started in step 2.14.
8. Dilute the cells in 5 mL of LB media to an OD_600_ of 0.01. Pour into a reservoir.
9. Add 80 µL of the diluted cells on to the drug linear dilutions prepared in step 3.6 using a multichannel micropipette. The final drug concentrations in each well is shown in Figure 7B. NOTE: The middle well in the dose-response will receive the serial IC50 for this drug. Seal plate to prevent evaporation.
10. Incubate the plate for 16 h at 37 °C.
11. Start two fresh bacterial cultures to use in step 4 (repeat steps 1.5-1.7).

### 4. Diagonal Drug Interaction Experiment

1. Measure the OD_600_ absorbance for linear dilution dose-response from step 3 (Figure 7B).
2. For each drug, choose the concentration that resulted in IC50 and prepare drug LEV, NAL and PNG in 100x IC50 concentrations.
3. Thaw fresh drugs, prepare 100x IC50 for each drug and prepare 1:1 drug mixtures by volume of LEV+NAL, LEV+PNG and NAL+PNG and 1:1:1 drug mixture by volume of LEV+NAL+PNG.
4. Prepare two plates for drug interaction experiments as shown in Figure 8.
5. Measure the OD_600_ of the 1:10 dilution of the cultures started in step 3.11.
6. Prepare OD_600_ = 0.01 dilutions of two cultures in two 10 mL of LB media.
7. Add 80 µL of the cells from culture 1 and 2 on plates 1 and 2, respectively. Seal the plates to prevent evaporation. Incubate the plates for 16 h at 37 °C.

### 5. Diagonal Drug Interaction Scores

1. Measure the OD_600_ absorbance for diagonal drug interaction experiment plates from step 4.
2. Normalize the growth by dividing the growth in each well with the growth in the no drug control well for each row.
3. For each row, locate the column that has growth inhibition closest to IC50, shown in orange in Figure 8 right. Assign IC50 based on the relative concentration of drug in this well.
4. For LEV+NAL, LEV+PNG and NAL+PNG and LEV+NAL+PNG dose-responses, calculate expected IC50 by averaging the IC50 of the single drugs in each combination. Note that averaging is a simple approximation for exact expected IC50 calculation as described before^12^.
5. Compute Fractional Inhibitory Concentration (FIC) scores by dividing the observed IC50 by the expected IC50 in each combination.

## Representative Results

Previously, we have reported the pairwise interactions among three drugs: LEV, NAL and PNG based on testing in miniaturized checkerboard assays, where two drugs were combined in a 4 x 4 matrix^13^,^14^. While NAL and LEV were synergistic, PNG was reported to be antagonistic with both LEV and NAL^13^,^14^. Here, we verified these pairwise interactions and measured the three-way interaction among these three drugs using a diagonal assay. Our results demonstrate that LEV+NAL+PNG is a synergistic 3-way antibiotic combination. Schematic representations for the results of the individual experimental procedure sections were given on the right side of Figure 7 and****Figure 8. Here, we present and interpret representative raw results from three plate readings, which are given in Figure 9. The top plate reading corresponds to serial and linear dilution experiments conducted in steps 2 and 3. The bottom two plate readings are duplicate interaction plates conducted in step 4.

The raw data in Figure 9 shows that top growth is around 0.55, but there is a 0.05 optical density of the media itself, as observed in the OD_600_ of the high drug concentrations where there is no growth. Therefore, we define IC50 as (0.55-0.05)/2 = 0.25. For each dose-response, the wells located closest to this value are shown with orange.

The upper half of Figure 9A shows the results from step 2, serial dose-response experiment. The IC50 wells for LEV is at column 10 in two replicates, which correspond to 4 ng/mL. The IC50 for NAL and PNG are at 3 µg/mL and 25 µg/mL, respectively. These concentrations correspond to the 1x concentration shown in Figure 7B. The lower half of Figure 9B shows the results from step 3, linear dose-response experiments. LEV, NAL and PNG's IC50 are found at 0.4x, 0.8x and 1.2x, respectively. These concentrations are assigned as the 1X IC50 for step 4.

Two plates corresponding to two replicate experiments are shown in Figure 9B, where the IC50 wells are shown with orange. In plate 1, all single drugs have their IC50 at 1x concentration. The expected IC50 for the pairwise or three-way combination is calculated by the arithmetic mean of constituent drugs, making expected IC50 for all combinations also 1x concentration. In plate 2, LEV and PNG have their IC50 at the 1x concentration, but NAL IC50 is at 1.2x. The expected IC50 for each combination is defined using the arithmetic means of these IC50 values. For example, the expected IC50 for LEV+NAL and NAL+PNG is 1.1x. The drug interaction score (FIC) for each combination is calculated by dividing observed IC50 with the expected IC50, as shown on the right side of the plates. Inspection of the FIC scores of the two plates demonstrates that LEV+NAL and LEV+NAL+PNG are synergistic, while LEV+PNG and NAL+PNG are antagonistic. FIC scores obtained in the two plates are in agreement, supporting the reliability of the protocol.

**Figure:**
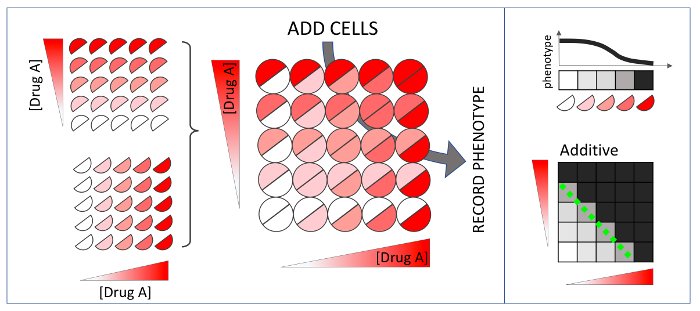

**Figure 1: Null model definition for Loewe additivity drug interaction model. **Two 5 x 5 matrices on a microplate, where drug A is linearly increased in one axis as shown at left, and a top concentration of drug inhibits a quantifiable phenotype (top right). The addition of these matrices is shown in the middle, where the lines connecting equipotent drug A concentrations in each single drug have the same concentration as drug A. When cells are added on this "checkerboard" of drug concentration combinations, it is expected that the recorded phenotype on this line will be equivalent for the wells it connects. In this self-self drug interaction experiment, the isobole depicting a phenotype (shown with a dashed green line) is expected to be linear, defining the additivity null model. Please click here to view a larger version of this figure.

**Figure:**
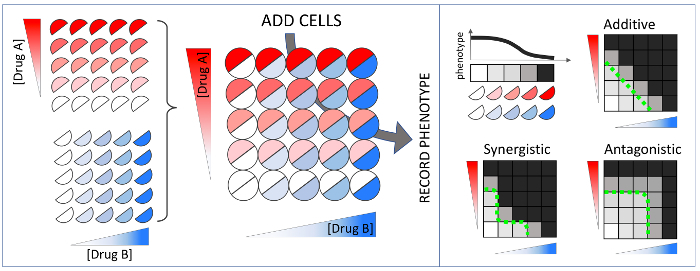

**Figure 2: Pairwise drug interactions according to Loewe additivity drug interaction model.** When two drugs are combined in a checkerboard assay as in Figure 1, the observed isophenotypic contour may be straight, convex or concave. At right, possible isophenotypic contours (dashed green lines) are superimposed on checkerboard assays. Drug combinations with straight isophenotypic contours are Loewe-additive, as the contours are not different from a self-self drug interaction, which is Loewe-additive by definition. When the isophenotypic contour is significantly concave or convex, the combination is synergistic or antagonistic, respectively. Please click here to view a larger version of this figure.

**Figure:**
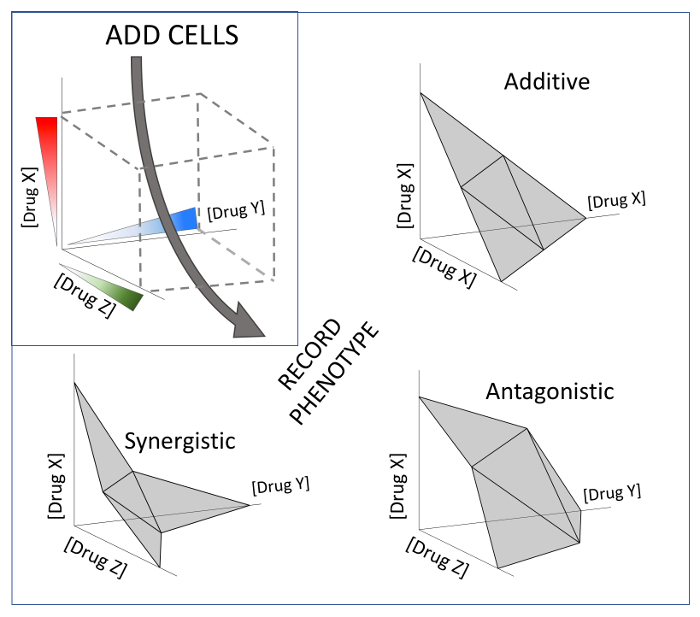

**Figure 3: Three-way drug interactions according to Loewe additivity drug interaction model.** Similar to the checkerboard assay for pairwise interactions, three-drugs are combined in a 3D grid (a "checkercube"), where each drug is linearly increased in one axis. If the three drugs were identical, the isophenotypic surface is expected to be flat, defining the additivity for three drug combinations. If the surface is more concave or convex than this Loewe-additive null model, drug combinations are synergistic or antagonistic, respectively. Please click here to view a larger version of this figure.

**Figure:**
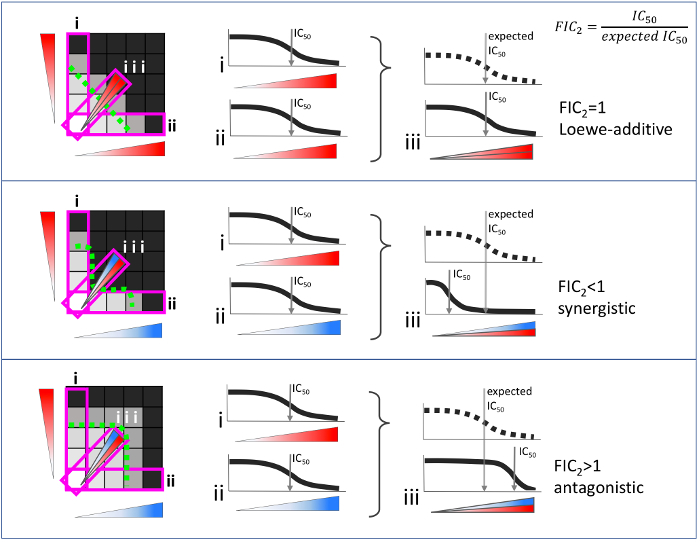

**Figure 4: Diagonal method to measure pairwise drug interactions.** For each checkerboard assay, only the regions shown in magenta rectangles are measured. i and ii are linearly increasing single drug concentrations. iii is assessed by making a 1:1 mixture of two drugs and linearly titrating this mixture as if it was a single drug. The FIC is equal to the observed IC50 in the combination divided by the expected IC50 of two single drugs. For the Loewe additivity model, the expected IC50 is approximated by the average IC50 of the two single drugs. A FIC value is 1 for Loewe-additive pairs and is lower or higher than 1 for synergistic or antagonistic pairs, respectively^2^,^12^. Please click here to view a larger version of this figure.

**Figure:**
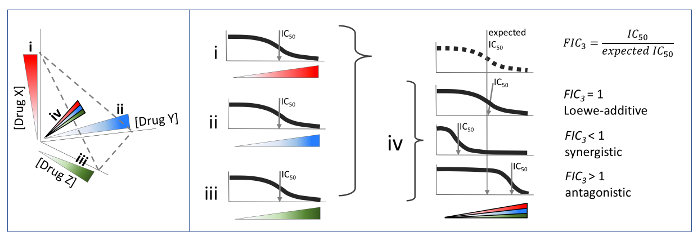

**Figure 5: Diagonal method to measure three-way drug interactions.** For each checkercube assay, only the regions shown are measured. i, ii and iii are linearly increasing single drug concentrations. iv is measured by making a 1:1:1 mixture of three drugs and linearly titrating this mixture as if it were a single drug. The Fractional Inhibitory Concentration is equal to the observed IC_50_ in the combination divided by the expected IC50 given three single drugs. Please click here to view a larger version of this figure.

**Figure:**
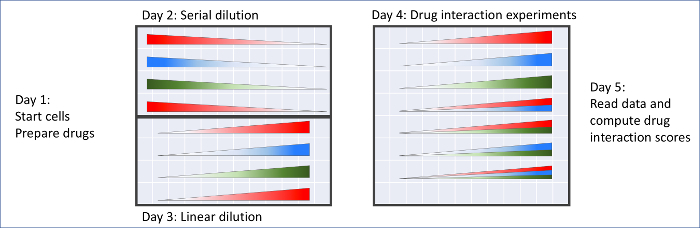

**Figure 6: Workflow for the diagonal method protocol described herein and the setup details for each microplate.** The plate shown in Day 4 is conducted in duplicate. Please click here to view a larger version of this figure.

**Figure:**
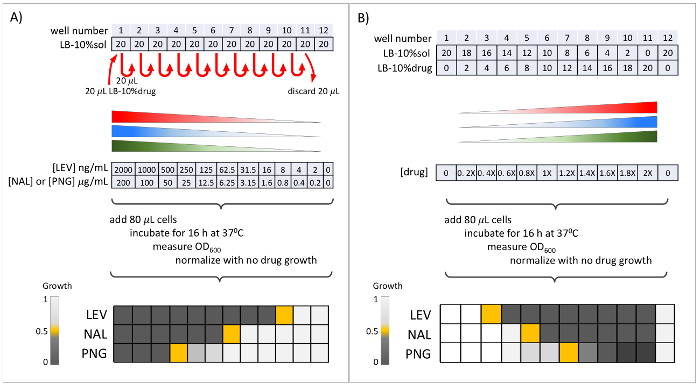

**Figure 7: Serial and linear dilution dose-response experiments.(A)** Preparation of serial dilution dose-response for one drug and corresponding final drug concentrations. *E. coli* cells are added to the plate; growth is recorded after 16 h. Serial IC50, shown in orange, is selected for each drug for use in the following day's linear dilution dose-response experiments. **(B)** Preparation of linear dilution dose-response for one drug and corresponding final drug concentrations. *E. coli* cells are added to the plate; growth is recorded after 16 h. For each drug, the selected IC50s that will be used in the following day's drug interaction experiments are shown in orange. Please click here to view a larger version of this figure.

**Figure:**
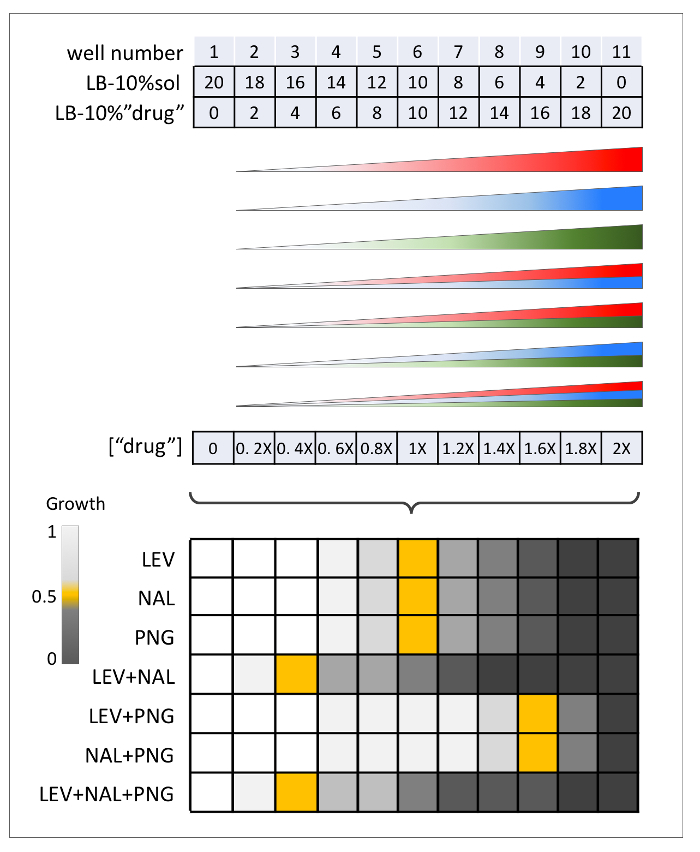

**Figure 8: Drug interaction experiments.** Preparation of interaction experiments is similar to single drug linear dose-responses, except that a 1:1 or 1:1:1 mixture of drugs is used for two or three drug dose-responses, respectively. Observed IC50s for each dose-response, depicted in orange, are used to calculate FIC scores. Please click here to view a larger version of this figure.

**Figure:**
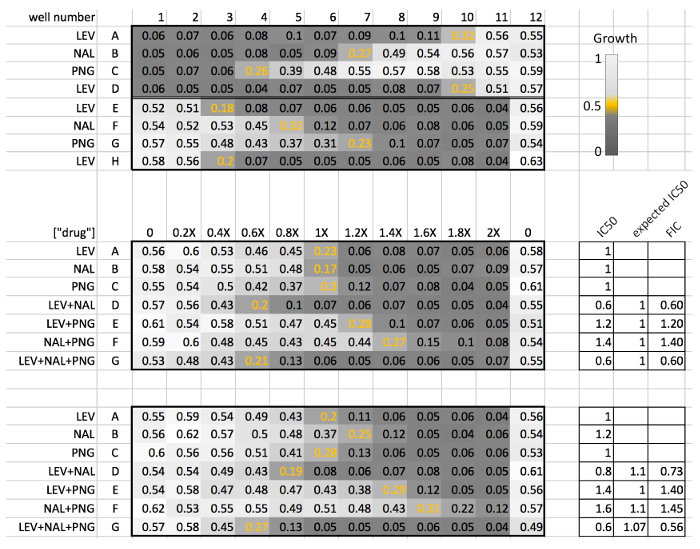

**Figure 9: Representative experiment results.** Representative results obtained using the described protocol are shown, with details provided in the main text. Please click here to view a larger version of this figure.

## Discussion

The use of drug combinations against pathogens or tumors is an attractive prospect, especially under the circumstances of the drying antibiotic pipeline. However, this potential is hampered by at least two difficulties. The first difficulty is the astronomical number of possible combinations. There are, for example, 4950 possible pairwise combinations among 100 antibiotics. All possible combinations among 100 antibiotics (2^100^) is on the same order of magnitude with the number of bacteria on Earth (~10^30^). How to predict strongly synergistic combinations among these possibilities has been the subject of numerous computational studies. The second difficulty is the measurement of high-order drug interactions. Consider that a computational platform may suggest that a certain 10-drug combination is strongly synergistic against a certain pathogen. Traditional methods to test drug interactions is too costly to verify or refute this hypothesis, therefore the study of synergy among many drugs has been outside the boundaries of scientific inquiry. The diagonal method, which was first proposed almost 30 years ago and was used in a few recent synergy screens provide a strong foundation for the first problem, by allowing testing of the interaction among many pairs. It solves the second problem by an informative sampling of the traditional assays and allows the study of high-order drug interactions.

We importantly note that our protocol uses a linear dosing for drug interaction measurements, to provide the sensitivity for detecting even weak interactions. Establishing the right concentration range for linear dosing is a challenging task. By first performing a serial dilution, we make an informed decision about the search space for linear dosing. However, the protocol can be modified to use 2-fold or higher serial dilutions for drug interaction testing. Such a modification would shorten the experiment time and allow the testing of more interactions; however, it would have sensitivity to detect only strongly synergistic or antagonistic interactions.

The protocol we described shows the measurement of pairwise or three-way interactions. A critical aspect of the protocol is that single agents are on the same plate as the combination, to minimize bias due to plate variations. Therefore, the protocol can be adjusted to measure interactions up to 7-way combinations by trivial modifications. Combinations of more than 7 drugs will require more than one 96-well microplate and additional considerations must be taken to ensure correct data integration, such as inter-plate replicates.

A notable limitation of the diagonal method is the restriction that each drug in the assay must inhibit the phenotype of interest. Therefore, the diagonal method is not useful for understanding the interactions among active agents and inert adjuvants. Such 'potentiating' interactions may be studied under alternative models such as Bliss or Highest Single Agent models.

An important consideration for the analysis of high-order drug interactions is the null model choice for the "expected IC50." When two drugs are combined, the combination's effect can only be compared to the single drug effects. When three drugs are combined, the combination's effect can be compared to the single effects or pairwise effects. For example, if all pairwise combinations of three drugs are synergistic, then it may be expected that these drugs will show a three-way synergy. A three-way interaction's deviation from what is expected from pairwise interactions has been recently dubbed "emergent interaction"^16^,^17^. For simplicity, our protocol describes the measurement of the three-way combination's "net interaction," which defines the null model as single drug effects. However, the data that is obtained from the protocol may also be used to compute the emergent interaction of the three-way combination. In our analysis, we defined the expected IC50 of the three-way combination as the average of the single drug IC50s. Alternatively, the expected IC50 can be defined as the average of the IC50s of pairwise combinations (~1.1-1.2). When the observed IC50 is divided by this alternative expected IC50, the obtained FIC provides the emergent FIC for the three-way combination, as previously described^12^. This consideration reveals that LEV+NAL+PNG is more synergistic than what would be expected from the pairwise interactions among three drugs, demonstrating that LEV+NAL+PNG has emergent synergy.

## Disclosures

The authors have nothing to disclose.

## References

[B0] Zimmermann GR, Lehar J, Keith CT (2007). Multi-target therapeutics: when the whole is greater than the sum of the parts. Drug Discovery Today.

[B1] Berenbaum MC (1989). What is synergy?. Pharmacological Reviews.

[B2] Cokol M (2013). Drugs and their interactions. Current Drug Discovery Technologies.

[B3] Yeh PJ, Hegreness MJ, Aiden AP, Kishony R (2009). Drug interactions and the evolution of antibiotic resistance. Nature Reviews Microbiology.

[B4] Lehár J, Krueger A, Zimmermann G, Borisy A (2008). High-order combination effects and biological robustness. Molecular Systems Biology.

[B5] Loewe S (1953). The problem of synergism and antagonism of combined drugs. Arzneimittelforschung.

[B6] Foucquier J, Guedj M (2015). Analysis of drug combinations: current methodological landscape. Pharmacology Research & Perspectives.

[B7] Wood KB (2016). Pairwise interactions and the battle against combinatorics in multidrug therapies. Proceedings of the National Academy of Sciences.

[B8] Berenbaum MC (1978). A method for testing for synergy with any number of agents. Journal of Infectious Diseases.

[B9] Weinstein ZB, Zaman MH (2017). Quantitative bioassay to identify antimicrobial drugs through drug interaction fingerprint analysis. Scientific Reports.

[B10] Horn T (2016). High-order drug combinations are required to effectively kill colorectal cancer cells. Cancer Research.

[B11] Cokol M, Kuru N, Bicak E, Larkins-Ford J, Aldridge BB (2017). Efficient measurement and factorization of high-order drug interactions in Mycobacterium tuberculosis. Science Advances.

[B12] Chandrasekaran S, Cokol-Cakmak M, Sahin N, Yilancioglu K, Kazan H, Collins JJ, Cokol M (2016). Chemogenomics and orthology-based design of antibiotic combination therapies. Molecular Systems Biology.

[B13] Mason DJ (2017). Prediction of antibiotic interactions using descriptors derived from molecular structure. Journal of Medicinal Chemistry.

[B14] Yilancioglu K (2014). Target-independent prediction of drug synergies using only drug lipophilicity. Journal of Chemical Information and Modeling.

[B15] Beppler C (2016). Uncovering emergent interactions in three-way combinations of stressors. Journal of the Royal Society Interface.

[B16] Tekin E, Beppler C, White C, Mao Z, Savage VM, Yeh PJ (2016). Enhanced identification of synergistic and antagonistic emergent interactions among three or more drugs. Journal of The Royal Society Interface.

